# Vigilance decrements in air traffic control as an aviation safety risk: a systematic review of modeling and prediction approaches

**DOI:** 10.3389/fnrgo.2026.1874304

**Published:** 2026-08-13

**Authors:** Manal Aich, Jamal El Aoufi, Ahmed Moussa

**Affiliations:** 1 Systems and Data Engineering Team (IDS Team), National School of Applied Sciences-Tangier, Abdelmalek Essaâdi University (UAE), Tangier, Morocco; 2 Mohammed VI International Academy of Civil Aviation, Nouaceur, Nouaceur Province, Morocco

**Keywords:** air traffic control, cognitive workload, EEG, eye tracking, fatigue detection, human factors, machine learning, neuroergonomics

## Abstract

**Introduction:**

Vigilance decrements in air traffic control (ATC) are a persistent problem for neuroergonomics: human cognitive performance must remain reliable across long operational shifts in an environment where lapses carry serious consequences. Sustained attentional failure is associated with degraded conflict detection, slower response to critical events, and airspace management errors. Impairment is bidirectional: underloaded controllers disengage while overloaded ones lose resolution, and current monitoring systems do not reliably distinguish between these states.

**Methods:**

This paper reports a systematic review of vigilance modeling and prediction in ATC, covering 34 articles from three major databases (PubMed, Scopus, Web of Science; initial search January 2025, forward citation update through February 2026), selected through pre-specified inclusion criteria following PRISMA 2020 guidelines.

**Results:**

The review addresses physiological and behavioral predictors, machine learning detection models, and multimodal fusion approaches for vigilance prediction. Task demand, time on position, circadian phase, and automation-induced passivity all shape vigilance in real operations, and none acts independently; existing predictive frameworks have not captured this structure well.

**Discussion:**

Each approach is evaluated against the constraints of real-time deployment, and the distance between controlled laboratory findings and tools usable on an operational floor remains substantial.

## 1 Introduction

In air traffic control, sustained attention is the core task. Controllers who lose vigilance miss conflicts, misread airspace dynamics, and respond late to time-critical events; these failures appear consistently across incident and accident records (Wahlgren, 2023; Pothana et al., 2025; Karim et al., 2024; Alharasees, 2025). The conditions that produce them vary: fatigue, monotony, circadian misalignment, and high cognitive load can each degrade performance, sometimes without the controller or supervisor noticing. The operational environment does not help. In high-density sectors, controllers simultaneously track radar returns, monitor aircraft trajectories, manage pilot communications, integrate meteorological data, and coordinate with adjacent sectors. That sustained parallel demand makes vigilance harder to maintain and lapses harder to detect.

Attentional failures in ATC take several forms: reduced alertness during low-traffic periods, degraded processing under workload spikes, slower response when automation handles routine tasks, and coordination errors under time pressure (McGee et al., 1997). The operational setting is physically static, controllers sit, watch screens, and talk, but the cognitive load is sustained across shifts that regularly exceed 6 h. The vigilance literature has consistently shown that this profile, low physical demand combined with unrelenting attentional requirements, produces reliable decrements in detection performance (Kahl, 2024).

Vigilance failure poses a specific problem for safety investigation. It leaves no artifact: no log deviation, no anomaly in the communication record, nothing that survives the event in a recoverable form. Incident reports and ATC logs document outcomes; they cannot reconstruct where a controller's attention was in the seconds before a near-miss. Real-time monitoring could change that, but it has not been integrated into operational safety systems in any systematic way.

Vigilance, workload, and fatigue are often used interchangeably, but the distinctions matter for measurement. Vigilance concerns sustained signal detection, catching a relevant event after an extended period of monitoring. Workload is the cognitive demand a task imposes at a given moment, regardless of duration. Fatigue is a cumulative reduction in physiological or cognitive capacity that degrades both (Borghini et al., 2014; Hemmerich et al., 2025). Comparative reviews of mental fatigue assessment tools report similar measurement challenges outside ATC (Kunasegaran et al., 2023). Treating these as equivalent produces models that cannot locate the source of a performance decrement.

Vigilance is the organizing construct for this review for three reasons. First, sustained signal detection is the primary cognitive task in ATC: controllers must catch rare, safety-critical events against a continuous background of routine traffic. Workload and fatigue affect that capacity but are not equivalent to it. Second, vigilance is the most tractable target for real-time physiological monitoring: decrements produce measurable signatures, theta power increases, alpha rebound, blink-rate changes, detectable in principle before a performance error occurs. Workload is better characterized through task-imposed demands than through operator state; fatigue is more effectively addressed through scheduling. Third, vigilance failure is specifically implicated in the detection gaps and delayed responses documented in ATC incident records, which makes it the construct most directly tied to the safety events this review is concerned with.

Vigilance measurement draws on three categories of evidence: subjective ratings, performance metrics, and physiological signals. Of these, physiological measures, EEG, ECG, electrodermal activity, blink rate, pupil dilation, are the most useful for continuous monitoring because they track cognitive state without requiring the controller to stop and report (Stuldreher et al., 2023). Machine learning models extract structure from these signals more effectively than conventional statistical approaches, particularly when data from multiple sensors are combined (Jin et al., 2025). Beyond detection, pairing physiological monitoring with active engagement strategies, such as embedding challenge elements into a task, can directly enhance vigilance rather than simply flagging its decline (Bodala et al., 2016).

This review has four objectives: mapping the experimental designs, simulation settings, and task paradigms used to study vigilance in ATC; comparing subjective, behavioral, physiological, and multimodal measurement approaches on sensitivity and practical deployability; evaluating machine learning and deep learning methods for vigilance prediction, with particular attention to models tested outside controlled laboratory settings; and identifying the methodological requirements a real-time, non-invasive monitoring system would need to meet to be usable in live operations.

## 2 Materials and methods

### 2.1 Review question and PICOS framework

The review was structured around the following question: What physiological, behavioral, and computational approaches have been used to model and predict vigilance decrements in air traffic control, and how well do these approaches meet the requirements for real-time operational deployment? The research question was defined using the PICOS framework as follows.

**Population:** Professional air traffic controllers and trainee controllers operating in simulated or live ATC environments, including en-route, remote digital tower, and emerging urban air mobility contexts.

**Interventions:** Measurement and modeling approaches targeting vigilance or sustained attention, including neurophysiological signal acquisition (EEG, ECG, HRV), ocular and behavioral monitoring (eye tracking, reaction time, facial analysis), and computational prediction models (statistical methods, machine learning, deep learning, and multimodal fusion).

**Comparators:** No single active comparator was specified. Studies were compared across measurement modalities (single-modality vs. multimodal), model architectures (classical machine learning vs. deep learning), and experimental settings (laboratory simulation vs. live operational shifts).

**Outcomes:** Primary outcomes were vigilance classification accuracy, detection sensitivity, and generalizability across participants and operational contexts. Secondary outcomes included sensor practicality for live deployment, individual calibration requirements, and the presence of closed-loop or intervention-ready system designs.

**Study designs:** Peer-reviewed experimental simulations, observational shift studies, and computational model development studies. Conference proceedings from established venues were included. Review articles, meta-analyses, editorials, and non-peer-reviewed preprints were excluded.

### 2.2 Literature search strategy

Three databases were searched: PubMed, Scopus, and Web of Science Core Collection, selected for their complementary coverage of biomedical, engineering, and human factors literature. The search combined terms across three axes: vigilance constructs (*vigilance, sustained attention, alertness, attention decrement*), operational context (*air traffic control, ATC, air traffic controller, aviation, airspace management*), and measurement or prediction methods (*physio* ^**^, *EEG, electroencephalogram, ECG, heart rate, HRV, eye tracking, pupil, blink, performance, reaction time, machine learning, deep learning, prediction*). Terms were searched in Title, Abstract, and Keywords fields; where keyword field searching was unavailable, Title and Abstract fields were used.

The initial database search was conducted in January, 2025. During the writing and revision period, a forward citation update was conducted through February, 2026 using database alert functions and manual citation tracking from included studies. Studies identified through this update were assessed against the same eligibility criteria as the primary search. Studies published after the initial search date, including all 2026-dated studies, were identified through this disclosed update process; 2025-dated studies already indexed at the time of the initial search were captured by it. Full, database-specific search strings, including Boolean syntax, field codes, date limits, and document type filters, are provided in Section 2.2.1 below.

#### 2.2.1 Full search strings

The following search strings were applied verbatim. The same Boolean logic was reapplied with an added date filter (after 15 January, 2025) for the forward citation update.

PubMed (initial search 15 January, 2025; forward citation update 25 February, 2026; fields: Title/Abstract [tiab]; language: English; document types: all, reviews excluded at screening):

((vigilance[tiab]  OR  “sustained
attention”[tiab]  OR  alertness[tiab] OR
“attention decrement”[tiab])
   AND (“air traffic control”[tiab] OR
ATC[tiab]  OR  “air traffic controller”[tiab]
   OR aviation[tiab]  OR  “airspace
management”[tiab])
   AND (EEG[tiab] OR
electroencephalogram[tiab] OR
electroencephalography[tiab]
   OR ECG[tiab]  OR  “heart rate”[tiab] OR
HRV[tiab]  OR  “heart rate variability”[tiab]
   OR “eye tracking”[tiab] OR
“eye-tracking”[tiab]  OR  pupil[tiab]
OR blink[tiab]
   OR “reaction time”[tiab]  OR  “machine
learning”[tiab] OR
“deep learning”[tiab]
   OR prediction[tiab] OR
physiology[tiab]  OR  physiological[tiab]))

*Records retrieved: 320*

Scopus (initial search 16 January, 2025; forward citation update 26 February, 2026; fields: TITLE-ABS-KEY; language: English; document types: Article, Conference Paper):

TITLE-ABS-KEY(
  (vigilance  OR  “sustained attention”  OR 
alertness OR “attention decrement”)
   AND (“air traffic control”  OR  ATC  OR  “air
traffic controller”
   OR aviation  OR  “airspace management”)
   AND (EEG  OR  electroencephalogram  OR  electroencephalography
   OR ECG  OR  “heart rate”  OR  HRV  OR  “heart
rate variability”
   OR “eye tracking”  OR  “eye-tracking”  OR 
pupil  OR  blink
   OR “reaction time”  OR  “machine
learning”  OR  “deep learning”
   OR prediction  OR  physio^*^))
   AND LIMIT-TO(LANGUAGE, “English”)
   AND (LIMIT-TO(DOCTYPE, “ar”) OR
LIMIT-TO(DOCTYPE, “cp”))

*Records retrieved: 250*

Web of Science Core Collection (initial search 17 January, 2025; forward citation update 27 February, 2026; fields: Topic [TS]; language: English; document types: Article, Proceedings Paper):

TS=((vigilance OR “sustained attention”  OR
alertness  OR  “attention decrement”)
   AND (“air traffic control”  OR  ATC  OR
“air traffic controller”
   OR aviation  OR  “airspace
management”)
   AND (EEG  OR  electroencephalogram  OR  electroencephalography
   OR ECG  OR  “heart rate”  OR  HRV  OR
“heart rate variability”
   OR “eye tracking”  OR  “eye-tracking”
OR pupil  OR  blink
   OR “reaction time”  OR  “machine
learning”  OR  “deep learning”
   OR prediction  OR  physio^*^))
   AND LA=English AND DT=(Article OR
“Proceedings Paper”)

*Records retrieved: 280*.

### 2.3 Procedure and eligibility criteria

**Figure 1:**
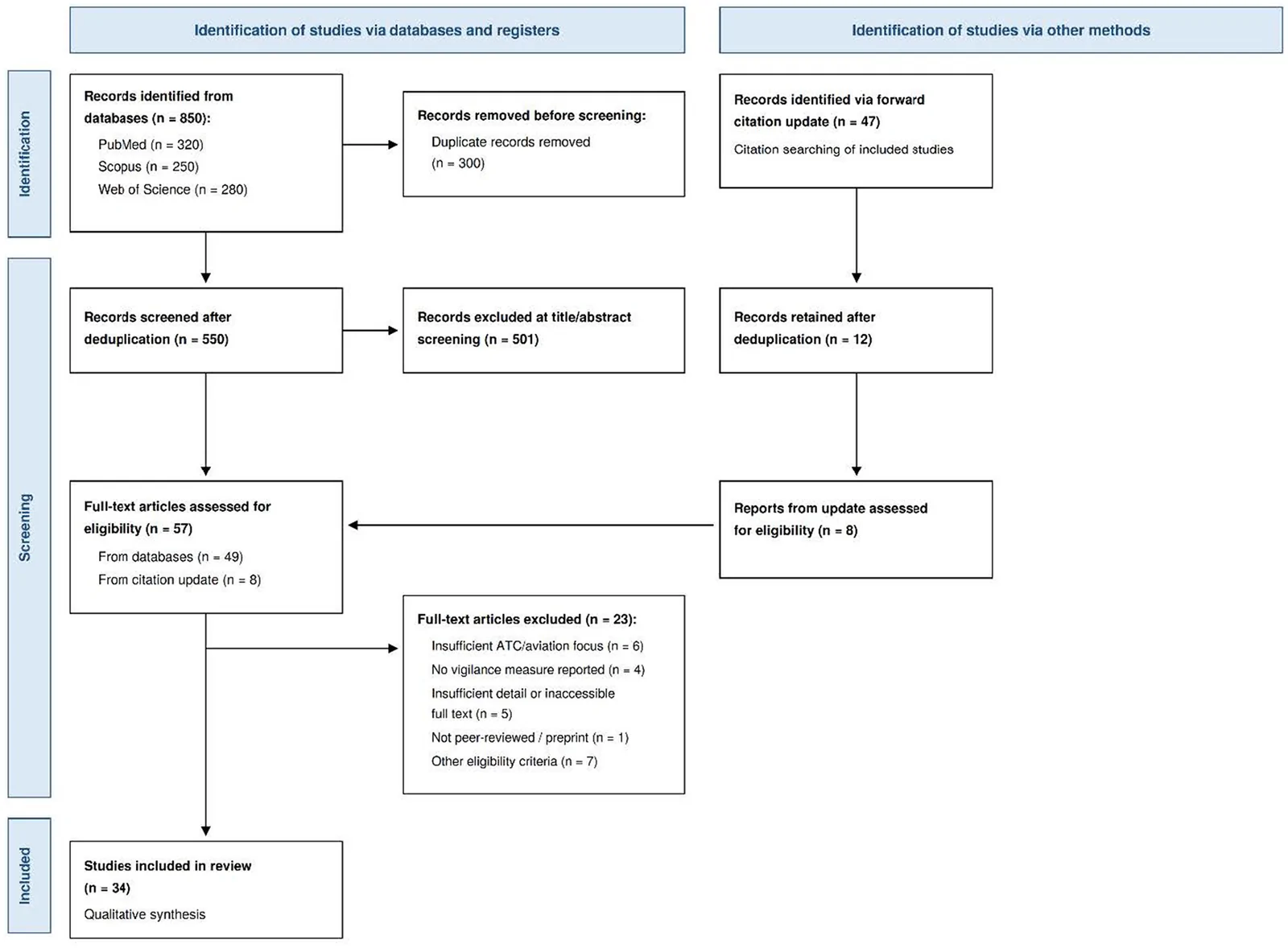
PRISMA 2020 flow diagram for the systematic review of vigilance modeling in ATC.

Study selection followed PRISMA 2020 guidelines (Page et al., 2021) (Figure 1). Database searches returned 850 records (320 PubMed, 250 Scopus, 280 Web of Science), of which 550 remained after deduplication. The forward citation update identified 47 additional candidate records; 12 were retained after deduplication and 8 advanced to full-text review. Title and abstract screening excluded 501 records. The remaining 57 full-text articles were assessed against Table 1; 23 were excluded. Thirty-four studies were included in the final synthesis.

**Table 1:** Inclusion and exclusion criteria.

Inclusion criteria	Exclusion criteria
(1) Written in English.	(1) Review papers, meta-analyses, editorials, or short communications.
(2) Published in peer-reviewed journals or reputed conference proceedings.	(2) Studies focusing solely on physical workload without cognitive or vigilance aspects.
(3) Examined vigilance, sustained attention, or alertness in operational or simulated environments.	(3) Studies not related to air traffic control, aviation, or similar safety-critical monitoring tasks.
(4) Utilized at least one measurement modality (e.g., EEG, ECG, eye-tracking, or behavioral metrics like reaction time).	(4) Studies not involving relevant measurement techniques for vigilance assessment.
(5) Included air traffic control tasks, aviation scenarios, or closely related monitoring-based tasks.	(5) Studies conducted on clinical populations or subjects with neurological, psychological, or sleep disorders affecting vigilance.
(6) Investigated modeling, detection, or prediction approaches, including statistical, machine learning, or deep learning methods.	(6) Studies lacking sufficient methodological details or without accessible full text.

Title and abstract screening was conducted by the first author (MA), with a 20% random sample independently screened by the third author (AM); disagreements were resolved by discussion with the second author (JEA) as arbitrator. Full-text review was conducted independently by MA and JEA, with all disagreements resolved by consensus. Inter-rater agreement for the 20% sample at title/abstract stage was κ = 0.81 (strong agreement). Figure 1 provides the PRISMA 2020 flow diagram including reasons for full-text exclusion with counts by reason.

### 2.4 Quality appraisal and risk-of-bias assessment

Quality appraisal was applied to all 34 included studies using a domain-adapted checklist reflecting the range of study designs: experimental simulations, observational shift studies, case studies, and computational model development. Six domains were assessed: (D1) objective and research question; (D2) participant selection and sample characteristics; (D3) ATC task or simulation description; (D4) vigilance or workload measurement validity; (D5) model development and validation procedures; (D6) reporting of limitations and generalizability. Each domain was rated Yes (Y), Partial (P), No (N), or Not Applicable (NA).

Studies rated Y on five or six domains are classified High quality; those with three or four Y ratings, Medium; those with fewer than three Y ratings, Low (consistent with the Y-count rule applied in Table 2). The full checklist with sub-items for each domain is provided in Section 2.4.1. Per-study domain ratings for all 34 included studies are in Table 2 (Section 2.4.2). Quality appraisal was conducted independently by MA and JEA; disagreements were resolved by discussion with AM. Cohen's kappa across all domain ratings: κ = 0.76 (substantial agreement). Of the 34 studies, 17 were rated High quality, 15 Medium, and 2 Low. Appraisal results informed interpretation; they were not used to exclude studies after eligibility screening.

**Table 2:** Per-study quality appraisal ratings (D1–D6).

References	D1	D2	D3	D4	D5	D6	Domains met	Classification
Aricò et al., 2016	Y	P	Y	Y	Y	P	**4**	Medium
Li et al., 2025	Y	Y	Y	Y	Y	Y	**6**	High
Peukert et al., 2025	Y	Y	Y	Y	Y	Y	**6**	High
Badea et al., 2025	Y	P	Y	P	P	Y	**3**	Medium
Zhong et al., 2025	Y	Y	Y	Y	Y	Y	**6**	High
Lemetti et al., 2025	Y	P	Y	Y	Y	Y	**5**	High
Terenzi et al., 2024	Y	Y	Y	Y	P	Y	**5**	High
Shao et al., 2024	Y	Y	Y	Y	Y	Y	**6**	High
Guo and Guan, 2025	Y	Y	Y	Y	Y	Y	**6**	High
Dasari et al., 2017	Y	P	Y	P	NA	Y	**3**	Medium
Li et al., 2023	Y	P	Y	Y	Y	P	**4**	Medium
Li et al., 2021	Y	P	P	Y	NA	Y	**3**	Medium
Li et al., 2024	Y	P	Y	Y	Y	Y	**5**	High
Jin et al., 2023	Y	Y	Y	Y	Y	Y	**6**	High
Hu et al., 2022	Y	P	Y	Y	Y	P	**4**	Medium
Farha et al., 2022	Y	P	Y	Y	Y	Y	**5**	High
Kamrud et al., 2021	Y	P	P	Y	Y	P	**3**	Medium
Migliorini et al., 2022	Y	P	Y	Y	P	Y	**4**	Medium
Brookings et al., 1996	Y	P	Y	Y	NA	P	**3**	Medium
Wang et al., 2021	Y	P	Y	Y	P	Y	**4**	Medium
Karimi-Rouzbahani et al., 2021	Y	Y	P	Y	Y	Y	**5**	High
Radnütz et al., 2020	Y	Y	Y	Y	NA	P	**4**	Medium
Liu et al., 2026	Y	P	Y	Y	Y	P	**4**	Medium
Wang et al., 2023	Y	P	P	Y	Y	P	**3**	Medium
Xu et al., 2024	Y	P	P	P	P	P	**1**	Low
Tan et al., 2025	Y	P	Y	Y	Y	Y	**5**	High
Hu et al., 2024	Y	P	P	Y	P	Y	**3**	Medium
Kouba et al., 2023	Y	P	P	Y	NA	P	**2**	Low
Pan et al., 2025	Y	Y	Y	Y	Y	Y	**6**	High
Gunzelmann et al., 2009	Y	Y	P	Y	Y	Y	**5**	High
Han et al., 2026	Y	Y	Y	Y	Y	Y	**6**	High
Sebastiani et al., 2020	Y	Y	Y	Y	Y	Y	**6**	High
Yu et al., 2023	Y	P	Y	Y	Y	Y	**5**	High
Di Flumeri et al., 2019	Y	P	Y	Y	P	Y	**4**	Medium

Inter-rater agreement: κ = 0.76 across all domain ratings (MA and JEA, independently); disagreements resolved with AM.

#### 2.4.1 Quality appraisal checklist, sub-items by domain

The following sub-items were assessed for each domain. Threshold rules for Y / P / N classification are stated after each domain.

D1—Objective and Research Question: (1.1) Study objective clearly stated; (1.2) vigilance or workload research question explicitly defined; (1.3) constructs (vigilance, workload, fatigue) clearly distinguished. Y = all three met; P = two met; N = fewer than two.

D2—Participant Selection and Sample Characteristics: (2.1) Inclusion/exclusion criteria specified; (2.2) sample size reported and justified; (2.3) participant characteristics reported (age, experience, professional vs. student); (2.4) recruitment method described. Y = 2.1 and 2.3 met plus at least one of 2.2 or 2.4; P = 2.1 and 2.3 only; N = fewer than two met.

D3—ATC Task or Simulation Description: (3.1) Task or simulation described in sufficient detail; (3.2) task duration reported; (3.3) traffic scenario or workload level described; (3.4) simulation fidelity or use of live data stated. Y = all four met; P = two or three met; N = fewer than two.

D4—Vigilance or Workload Measurement Validity: (4.1) At least one validated modality used; (4.2) ground truth definition explicitly stated; (4.3) extracted features described; (4.4) vigilance manipulation method described. Y = 4.1 and 4.2 met plus at least one of 4.3 or 4.4; P = 4.1 met but 4.2 absent or unclear; N = 4.1 not met.

D5—Model Development and Validation Procedures: (5.1) Modeling approach described in sufficient detail; (5.2) validation strategy specified (within-subject, cross-subject, *k*-fold, hold-out); (5.3) performance metrics reported; (5.4) risk of overfitting addressed. Y = all four met; P = 5.1 and 5.3 met but 5.2 or 5.4 absent; N = fewer than two met; NA = not a modeling study.

D6—Limitations and Generalizability: (6.1) Limitations explicitly discussed; (6.2) generalizability to operational ATC addressed; (6.3) future work provided. Y = all three met; P = two met; N = fewer than two.

#### 2.4.2 Per-study quality appraisal ratings

Table 2 shows domain-level ratings for all 34 included studies. Y = Yes (green); P = Partial (amber); *N* = No (red); NA = Not Applicable (gray). ‘Domains Met' counts Y ratings only. Classification thresholds: High ≥ 5 Y; Medium 3–4 Y; Low < 3 Y.

### 2.5 Data extraction

Data extraction used a structured sheet applied uniformly across all 34 studies, recording: study parameters, participant characteristics (including total *N*), task setting and duration, experimental paradigm, vigilance manipulation method, measurement modality, extracted features, modeling approach, validation strategy (within-subject/cross-subject/mixed), setting (simulator/field/laboratory), and performance metrics (accuracy, sensitivity, specificity, F1-score, false alarm rate where reported).

### 2.6 Transparency and openness

This review follows TOP guidelines and PRISMA 2020 reporting standards. The search strategy is documented in Sections 2.2 and 2.2.1; inclusion and exclusion criteria in Table 1; selection flow in Figure 1; quality appraisal checklist in Section 2.4.1; per-study ratings in Table 2. All sources are published peer-reviewed articles. The review was not pre-registered on PROSPERO prior to data collection; the authors acknowledge this as a methodological limitation that reduces protection against outcome reporting bias, and prospective registration is recommended for future reviews.

### 2.7 Publication trends and article categories

Roughly three in 10 included articles were published in 2025–2026 (29.4%), and most of the literature (58.8%) dates from 2023 onward; a small minority (11.8%) pre-date 2019. EEG and eye-tracking are the most common measurement modalities (58.8% and 50.0%, respectively), and a substantial share combine signals from multiple sources. China and Italy account for the largest share of deep learning studies. Approximately 65% of studies recruited professional ATCOs. Full distributions are in Table 3.

**Table 3:** Distribution of selected articles across study characteristics.

Category	Sub-category	*n*	%
Publication year	≤2018	4	11.8%
	2019–2022	10	29.4%
	2023–2024	10	29.4%
	2025–2026	10	29.4%
Measurement modality	EEG (neurophysiological)	20	58.8%
	Eye-tracking (ocular)	17	50.0%
	HRV/ECG (cardiovascular)	10	29.4%
	Multimodal fusion	15	44.1%
Study population	Professional ATCOs	22	64.7%
	Students/trainees	12	35.3%
Geographical distribution	China	15	44.1%
	Italy	5	14.7%
	USA	4	11.8%
	Sweden	3	8.8%
	Hong Kong	3	8.8%
	Others	4	11.8%
ATC environment	En-route/area control	25	73.5%
	Remote digital tower (RDT)	6	17.6%
	Future ops (UAM/eVTOL)	0	0.0%
	Laboratory/fundamental	3	8.8%

## 3 Results

### 3.1 Experimental paradigms and ATC environments

#### 3.1.1 Subject characteristics

Sample sizes ranged from 2 (Terenzi et al., 2024), a report of two case studies, to 57 (Li et al., 2021), with most studies recruiting between 10 and 30 participants. Five studies exceeded 30 participants: (Li et al., 2021) (*N* = 57), (Li et al., 2024) (*N* = 41), (Han et al., 2026) (*N* = 36), (Zhong et al., 2025) (*N* = 34), and (Pan et al., 2025) (*N* = 34). Per-study participant counts for the modeling studies are listed in the *N* column of Table 4, and counts for the remaining studies are reported inline where relevant. Approximately 65% of studies (22 of 34) recruited professional ATCOs, while the remaining 12 used student or trainee controllers (Table 3).

**Table 4:** Summary of ML/DL studies, with participant *N*, validation type, experimental setting, operational metrics, and evidence tier.

References	Problem	Modality/features	Model	*N*	Valida-tion	Setting	Best performance	Op. metrics	Tier
Di Flumeri et al. (2019)	Vigilance class	EEG/IAF-relative θ,α,β power	asSWLDA	14	WS	Sim.	Vigilance score 0.42 → 0.51 (*p* = 0.002)	N/R	1—PoC
Aricò et al. (2016)	Workload class. (closed-loop)	EEG/frontal θ, parietal α PSD	asSWLDA (online classifier)	12	WS	Sim.	75% Acc. (offline cal.)	N/R	1—PoC
Li et al. (2025)	Detection failures	Eye-tracking/gaze dynamics	Random forest	26	WS	Sim.	80% precision	Prec: 80%	1—PoC
Zhong et al. (2025)	Fatigue detection	EEG/spatio-temporal connectivity	ST-graph + center loss	34	WS	Sim.	96.73% Acc.	N/R	1—PoC
Lemetti et al. (2025)	Workload class.	Eye-tracking/58 features (reduced)	RF, SVM, KNN	18	WS	Sim.	86.0% Acc.	N/R	1 – PoC
Shao et al. (2024)	Workload class.	EEG/PSD/32-channel	SVM	30	WS	Sim.	88.5% Acc.	N/R	1—PoC
Guo and Guan (2025)	Work state Rec.	Facial landmarks/eye closure	Improved Xception	N/A	CS	Lab.	94.36% Acc.	N/R	2—CS
Li et al. (2023)	SA recognition	EEG + eye-tracking/power + gaze	BP Neural Network	15	WS	Sim.	84.1% Acc.	N/R	1—PoC
Li et al. (2024)	Workload class.	EEG/beta, theta, alpha/3 channels	mRMR + SVM	41	WS	Sim.	98.6–100%	N/R	1—PoC
Jin et al. (2023)	Forgetting pred.	Eye-tracking/saccade + fixation	CNN-LSTM	20	WS	Sim.	79.2% Acc.	N/R	1—PoC
Hu et al. (2022)	Fatigue detection	Eye + behavior/multiple features	Fuzzy Fusion	16	WS	Sim.	97.0% Acc.	N/R	1—PoC
Farha et al. (2022)	Vigilance class.	EEG + Eye-tracking/CCA fusion	SVM (CCA-based)	10	WS	Sim.	97.2% Acc.	N/R	1—PoC
Kamrud et al. (2021)	Vigilance decrement class.	EEG/5-band spectral power (MLPNN); raw time-series (TCN)	MLPNN (best); TCN-AE; TCN	14	Cross-task, L2PO-CV (7-fold)	Lab.	64% Bal. Acc. (95% CI 0.59–0.69)	AUROC: 0.71	1—PoC
Wang et al. (2023)	Vigilance class.	HRV/SDNN, RMSSD, LF/HF	Hidden Markov Model	25	WS	Lab.	>80% Acc.	N/R	1—PoC
Tan et al. (2025)	Fatigue detection	Facial images/Eye-closure/Mouth	FF-YOLO (YOLO11)	N/A	N/A	Sim.	94.2% mAP@50	mAP: 94.2%	1—PoC
Xu et al. (2024)	Fatigue class. (3-class)	Facial video + radiotelephony audio/dual-stream CNN fusion	AF Dual-Stream CNN	N/R	N/R	Field/Tower	98.03% Acc. (fused)	Facial-only: 96.0%; Audio-only: 62.88%	1—PoC
Hu et al. (2024)	Fatigue detection	Eye-tracking/fixation, saccade, blink, pupil	SVM; RF	11	Hold-out (75/25 SVM; 85/15 RF)	Sim.	67% Acc. (SVM)	RF: 65% Acc.	1—PoC
Pan et al. (2025)	SA prediction	Eye-tracking + HRV/Gaze entropy	LightGBM (TPE-Opt)	34	Mixed	Sim.	*R*^2^ = 0.7845	N/R	1—PoC
Han et al. (2026)	Fatigue detection	Ocular + Cardiac/Multimodal	Cost-sensitive XGBoost	36	CS	Sim.	83.52% Acc.	Sens./Spec. reported	2—CS
Yu et al. (2023)	Fatigue Rec.	EEG + Eye/CNN-LSTM (RecMF)	CNN-LSTM	15	WS	Sim.	Acc. not recoverable	N/R	1—PoC

WS, within-subject; CS, cross-subject; Mixed, partial cross-subject; Sim., simulator; Lab., laboratory/dataset; N/R, not reported; N/A, not applicable (system or dataset study). Tier 1. does not meet cross-subject deployability criteria, comprising both proof-of-concept detection studies (small-N, within-subject, simulator/laboratory-based) and system-, optimization-, or dataset-level studies (marked N/A for validation) that were not evaluated for cross-subject generalization; Tier 2, stronger external validity via cross-subject validation. Studies (Guo and Guan, 2025) and (Han et al., 2026) are classified as Tier 2 (cross-subject). No included study used live operational (field) shift data, and no study combined field data with cross-subject validation. For (Yu et al., 2023), the headline accuracy could not be recovered from the source paper because the reported figure is inconsistent across the text and tables; the study's validation procedure is nonetheless fully specified (defined multi-sensor fusion pipeline with a stated train/test split), which is why it retains a Y on D5 in Table 2. Its quality classification therefore reflects methodological reporting, not the recoverability of a single performance value. For (Di Flumeri et al., 2019), the EEG-based vigilance classifier (asSWLDA) is used within a closed-loop adaptive-automation system rather than evaluated as a standalone classifier; the source paper reports a group-level vigilance index comparison between conditions, not a held-out classification accuracy, sensitivity, or false-alarm rate, hence Tier 1 despite the closed-loop design (see Section 4.1).

#### 3.1.2 ATC task settings

Most studies were conducted in simulated environments (Aricò et al., 2016; Badea et al., 2025; Zhong et al., 2025; Lemetti et al., 2025; Shao et al., 2024; Dasari et al., 2017; Li et al., 2023, 2024; Jin et al., 2023; Hu et al., 2022; Farha et al., 2022; Migliorini et al., 2022; Wang et al., 2021; Liu et al., 2026; Tan et al., 2025; Pan et al., 2025; Han et al., 2026; Yu et al., 2023); four used data collected in real-shift or operationally realistic settings (Peukert et al., 2025; Terenzi et al., 2024; Li et al., 2021; Sebastiani et al., 2020); none, however, developed a predictive vigilance model validated on live operational data (see Section 4.1). En-route radar surveillance is the most frequent task setting, with six studies on Remote Digital Tower (RDT) environments (Li et al., 2025; Zhong et al., 2025; Shao et al., 2024; Dasari et al., 2017; Pan et al., 2025; Han et al., 2026).

#### 3.1.3 Vigilance and workload manipulation

Vigilance was most commonly manipulated through time-on-task: extended continuous monitoring periods in simulated or real operational settings (Zhong et al., 2025; Farha et al., 2022; Han et al., 2026; Yu et al., 2023). A smaller set induced fatigue through sleep deprivation or circadian disruption associated with shift scheduling (Peukert et al., 2025; Terenzi et al., 2024; Gunzelmann et al., 2009).

### 3.2 Measurement modalities and sensor integration

#### 3.2.1 Neurophysiological and cardiovascular devices

EEG was the most common modality, used in over 50% of studies (Zhong et al., 2025; Shao et al., 2024; Li et al., 2024; Farha et al., 2022; Kamrud et al., 2021; Migliorini et al., 2022; Sebastiani et al., 2020; Yu et al., 2023). One study used MEG (Karimi-Rouzbahani et al., 2021). Ten studies recorded cardiovascular signals via ECG or HRV (Table 3).

#### 3.2.2 Ocular tracking and computer vision systems

Eye-tracking was used in roughly 50.0% of studies (Li et al., 2023; Wang et al., 2021; Hu et al., 2024; Pan et al., 2025; Han et al., 2026). Three studies used camera-based computer vision: one applied the FF-YOLO algorithm (Tan et al., 2025), another used Vision-Language Models (VLMs) (Liu et al., 2026), and a third combined facial imagery with radiotelephony audio in a dual-stream network (Xu et al., 2024). Camera-based computer vision systems can be installed without attaching sensors to the controller; wearable eye-trackers, by contrast, require the controller to wear a device.

#### 3.2.3 Reference vigilance and workload measures

Three studies assessed subjective workload with the NASA-TLX (Badea et al., 2025; Wang et al., 2021; Hu et al., 2024); sleepiness was measured via the KSS or SSS in four studies (Peukert et al., 2025; Terenzi et al., 2024; Hu et al., 2024; Han et al., 2026). The PVT-B was used as a behavioral baseline in four studies (Peukert et al., 2025; Terenzi et al., 2024; Dasari et al., 2017; Han et al., 2026).

### 3.3 Feature engineering and vigilance indices

Twenty-six studies found measurable changes in physiological signals with vigilance level or time on task. EEG spectral features were the most common (Zhong et al., 2025; Shao et al., 2024; Li et al., 2024; Farha et al., 2022; Kamrud et al., 2021; Sebastiani et al., 2020; Yu et al., 2023): theta (4–8 Hz) and alpha (8–13 Hz) power increase as vigilance declines (Sebastiani et al., 2020), while beta (13–30 Hz) and gamma (30–45 Hz) activity is associated with higher alertness (Li et al., 2024). Gaze-based features appeared in 17 studies (Li et al., 2023; Wang et al., 2021; Hu et al., 2024; Pan et al., 2025; Han et al., 2026) and HR or HRV in 10 (Table 3).

Ocular and cardiac indices are less consistent than the EEG findings above in one respect: what changes, rather than how. Fixation duration, saccade dynamics, blink rate and duration, and pupil diameter are the ocular features that studies most often return to. Fixation duration proved the strongest single fatigue predictor when tested against saccade, blink, and pupil measures (Hu et al., 2024), and another study fed sequences of fixations and saccades directly into a model that predicted lapses in situational awareness (Jin et al., 2023). Gaze entropy, essentially how randomly a controller scans the display, and reduced attention to task-relevant areas both tracked declining situational awareness in remote tower simulations (Pan et al., 2025).

Cardiac features turn up less often, and when a direction of change is reported at all, it does not always agree across studies. SDNN and the LF/HF ratio rose while HF power fell as vigilance declined in one HRV study (Wang et al., 2023), the opposite of what the HRV-cognitive load literature usually reports outside aviation; the reviewed studies offer no explanation for the discrepancy. A multimodal study pairing gaze entropy with HRV found the two modalities complemented each other but did not break down which cardiac feature drove the effect (Han et al., 2026), and the LF/HF ratio also turned up as an important predictor of situational awareness in (Pan et al., 2025), without a stated direction either.

What this literature agrees on is which features to extract: fixation duration, saccade dynamics, blink rate, pupil diameter, and gaze entropy for the eyes; SDNN, RMSSD, and the LF/HF ratio for the heart. Agreement is far thinner when it comes to direction, magnitude, or threshold. Most studies report that a feature was predictive without saying whether it rose or fell with declining vigilance, and the one study that does report a direction (Wang et al., 2023) cuts against what is usually assumed elsewhere. That matters for deployment as much as theory: a monitoring system needs a known, stable direction of change to set an alerting threshold, and nothing in this literature shows that ocular or cardiac indices behave consistently enough across people and settings to support one without individual calibration, consistent with the deployability constraints discussed in Section 4.1.

### 3.4 Computational modeling and predictive performance

Twenty studies developed machine learning or deep learning models to classify or predict vigilance and workload states (Table 4). The feature selection and extraction methods these models rely on, including mRMR-based approaches (Table 4), belong to a broader methodological family benchmarked outside the vigilance literature (Nogales and Benalcazar, 2023). Comparing performance across studies is complicated by differences in experimental design, participant samples, task fidelity, sensor modalities, and validation strategy.

Meta-analysis was not conducted given the heterogeneity described above. Among studies reporting classification accuracy, values ranged from 64% to 100% (noting that Kamrud et al., 2021 reports 64% Balanced Accuracy and Li et al., 2024 reports 98.6–100% on a small within-subject sample). Models using CNN-LSTM architectures, spatio-temporal graphs, or multimodal fusion tended toward higher accuracy, with several exceeding 90%, but sample sizes were small, validation was mostly within-subject, and figures are not directly comparable across studies.

## 4 Discussion

### 4.1 Cross-study synthesis and primary implications for aviation safety

Prediction accuracy in the reviewed studies is often high, but the conditions that produced those results sharply limit what can be concluded. EEG, eye tracking, HRV, facial features, and multimodal fusion all show predictive signal; whether those results are reliable depends on sample size, task realism, ground truth definition, validation design, and sensor quality.

The findings are best read through a two-tier lens. Tier 1, proof-of-concept detection studies, consists of small-sample, within-subject, simulator-based experiments that demonstrate feasibility under controlled conditions. The majority of studies in this review fall into this tier. Tier 2, studies with stronger external validity, includes those using cross-subject validation, field data from operational shifts, or operationally relevant metrics such as sensitivity, specificity, and false alarm rate alongside accuracy. Three studies were initially flagged as candidate Tier 2 (Li et al., 2025; Guo and Guan, 2025; Han et al., 2026); however, on examination of the source papers none used live operational (field) data: (Li et al., 2025) is a controlled detection-failure induction experiment (within-subject), (Guo and Guan, 2025) is a facial-image dataset model, and (Han et al., 2026) is a high-fidelity simulation study whose cross-subject (leave-one-subject-out) performance dropped markedly. On this basis (Li et al., 2025) is reclassified as Tier 1, while (Guo and Guan, 2025) and (Han et al., 2026) are retained as Tier 2 on the strength of their cross-subject validation alone. No included study therefore provides combined field and cross-subject evidence. Conclusions about operational deployability should be drawn from Tier 2 studies only, and even these rest on dataset or simulation data rather than live operations; Tier 1 studies establish technical feasibility but do not establish readiness for deployment in live operations.

The same three problems appear repeatedly. Models achieve high accuracy on small or homogeneous samples, which limits generalizability. The sensors that produce the best laboratory results, high-density EEG arrays, precision eye trackers, are not the sensors that can be deployed during a live shift. And ground truth definitions vary enough across studies that a model trained on one definition may not transfer to another.

For a vigilance detection system to be considered operationally deployable, it should meet at minimum the following criteria: (1) cross-subject validation on a sample representative of the operational ATCO population; (2) field validation under real shift conditions, not only simulator data; (3) reporting of sensitivity and false alarm rate at operationally relevant classification thresholds, not only overall accuracy; and (4) a closed-loop design that specifies the action triggered by a detected decrement. None of the studies in this review simultaneously meets all four criteria. Two partial exceptions are worth noting: Aricò et al. (2016) and Di Flumeri et al. (2019) each implement a genuine closed-loop system, in which an EEG-based workload or vigilance index, computed with the same asSWLDA classifier family, directly and automatically changes the interface's level of automation once a workload or vigilance threshold is crossed, satisfying criterion (4) on its own. However, both systems were calibrated and validated within-subject rather than across subjects (*N* = 12 and *N* = 14, respectively), tested in high-fidelity simulators rather than under live shift conditions, and neither paper reports sensitivity or false-alarm rate for the underlying classifier; they therefore do not meet criteria (1)–(3), and the overall conclusion that no included study satisfies all four criteria simultaneously still holds.

### 4.2 Small sample sizes, statistical power, and overfitting risk

Small sample sizes are a recurring limitation. Models trained on data from a small number of controllers risk learning idiosyncratic patterns, a form of overfitting rarely detectable without independent validation samples.

Statistical power benchmarks put the sample sizes in context. For between-subjects comparisons at a medium effect size (Cohen's *d* ≈ 0.5) and 80% power, approximately 64 participants per group are required. Within-subject repeated-measures designs, used in most studies here, can reach adequate power for detecting main effects with smaller samples (around 20–30 participants for *d* ≈ 0.5), but cross-subject generalization requires substantially larger and more diverse recruitment. By these standards, the majority of studies (sample sizes 10–20, within-subject validation) are adequately powered for detecting physiological changes within their own sample, but not for cross-subject generalization claims. That distinction is not consistently drawn in the reviewed literature: several studies report high accuracy figures without noting that their validation design does not support generalization beyond the study sample.

Closing this gap requires larger and more diverse recruitment, repeated-measures designs, and validation across multiple facilities.

### 4.3 Simulator fidelity vs. real-world ATC operations

The substantial majority of studies used simulator environments, which allow controlled variation of traffic load and task complexity but do not replicate the accountability pressures, environmental noise, and system interdependencies of live operations (Kamrud et al., 2021). One included study (Li et al., 2021) is a notable exception, linking real-world ATCO alertness data to actual ATM occurrences and traffic volumes; however, it does not present a trained predictive or classification model, and so falls outside the Tier 1/Tier 2 framework applied to modeling studies in Table 4. A model trained on simulator data may not generalize to the conditions it would actually encounter in deployment.

The differences between simulated and real-world ATC environments most likely to affect model transfer are: (1) Cognitive and affective context, operational controllers work under real accountability pressure with career consequences for errors, altering arousal and baseline physiological profiles relative to simulated conditions; (2) Environmental noise, ATC facilities contain ambient sound from radio traffic and adjacent workstations absent from most simulations, affecting EEG signal quality, ocular baseline, and HRV; (3) Traffic density and event timing, simulators present scripted traffic with known event rates, whereas real operations involve unpredictable distributions and genuine emergencies; (4) Technology and interface differences, certified ATC hardware differs from simulation interfaces in display resolution, interaction modality, and ergonomics, affecting gaze behavior that eye-tracking models depend on; (5) Fatigue accumulation, real operational fatigue builds across a full shift roster spanning multiple days and nights, whereas most simulator studies induce fatigue over a single session.

For sensor deployment in live ATC facilities, additional feasibility constraints apply: regulatory requirements for non-interference with ATC equipment (limiting sensor types and wireless protocols), hygiene requirements for shared workstations, operational continuity requirements that preclude setup procedures longer than a few minutes, and the need for controller consent and data governance frameworks for continuous physiological monitoring of certified personnel.

### 4.4 Evolution of measurement technologies

Gel-based EEG remains the most studied neurophysiological modality. Eye-tracking has shifted from head-mounted to remote configurations, with at least one study incorporating deep learning for contact-free fatigue detection (Tan et al., 2025), and another combining non-contact facial-video and radiotelephony-audio streams to avoid attaching sensors to the controller altogether (Xu et al., 2024). For operational deployment, sensor practicality is a more immediate constraint than algorithmic performance.

However, while no included study used dry-electrode EEG under ATC conditions, dry-electrode systems involve important signal quality trade-offs that are relevant to any future deployment. Dry electrodes eliminate conductive gel but introduce substantially higher contact impedance, reducing signal-to-noise ratio, particularly for delta and theta power, the features most central to vigilance detection. They are also more susceptible to motion artifact over extended sessions. Electrode-skin coupling in dry systems degrades progressively during a shift as perspiration accumulates, potentially altering the vigilance features extracted later in a shift relative to those at shift onset. These constraints mean that dry-electrode systems currently require more aggressive artifact rejection and adaptive re-referencing to approach the signal quality of gel-based systems on vigilance-sensitive features, a gap not yet systematically characterized under ATC-relevant conditions. Future research should explicitly compare dry- and gel-electrode classification performance on vigilance features under extended, ATC-representative session durations.

### 4.5 Future considerations: from detection to accident prevention

Current models classify vigilance state; the harder and more operationally consequential problem is what a system should do when a decrement is detected. A closed-loop vigilance monitoring system, as defined here, is one in which a real-time classifier drives an automated or supervisory response rather than simply logging a state label. Such responses might include triggering a supervisor alert, adjusting automation support (for example, increasing conflict alert sensitivity), modifying task allocation by redirecting incoming traffic, or initiating a structured controller handover protocol. Proactive monitoring and alert-system design principles from complex data ecosystems outside aviation (Adepoju et al., 2022) may offer transferable lessons for this closed-loop design problem. Closed-loop designs have been demonstrated in adjacent domains, notably EEG-based driver fatigue and lapse detection-and-mitigation systems that issue adaptive alerts or trigger corrective control (Huang et al., 2016; Jung et al., 2014), frameworks for balancing automation and worker safety exist in other high-consequence industries such as oil, gas, and renewables (Egbumokei et al., 2024), but closed-loop vigilance systems remain largely absent from ATC. Translating detection into intervention requires not only a reliable classifier but a model of the intervention-response relationship: what action, triggered at what classification threshold, produces the safety benefit without disrupting normal operations? This is a system design problem, not only a signal processing problem.

A vigilance alert that cannot be interpreted is unlikely to be acted on. Operational supervisors and sector managers need to understand which physiological or behavioral features drove a classification decision. Explainable AI techniques (SHAP values, attention maps, feature attribution methods) have been applied in adjacent occupational monitoring contexts and should be incorporated into vigilance detection system design from the outset, not retrofitted after classifier development. A comparable pattern holds outside aviation: AI diagnostic tools in industrial fault management improve operator performance only when their outputs remain interpretable enough for operators to catch incorrect recommendations (Ung, 2025).

Vigilance profiles differ substantially across controllers in circadian pattern, workload tolerance, gaze behavior, and physiological response to fatigue. A model optimized on group averages may miss individual-level degradation precisely where early detection matters most. Individual calibration, person-specific baseline models updated over time, is a design requirement for any system intended to monitor individual controllers in real operations, requiring both adaptive modeling capability and a data governance framework for managing individual physiological baselines across shift rosters. Human-centric workload assessment frameworks developed for other high-precision domains, such as maintenance and assembly, offer a template for this kind of individualized monitoring (Vitti et al., 2025).

No study in this review validated a vigilance model under real shift conditions with a sample large enough to support generalization. Field validation requires coordinated access to operational ATC facilities, regulatory engagement, and study designs that capture the full range of controller states and traffic conditions encountered in real operations. This cannot be accomplished in simulation alone.

The pathway from research prototype to certified operational monitoring tool involves regulatory requirements that go beyond technical performance: non-interference with certified ATC equipment, hygiene and ergonomic requirements for shared workstations, data protection frameworks for continuous biometric monitoring of workers, and compatibility with occupational health regulation. Engaging with aviation regulatory bodies (EASA, FAA, ICAO) and national data protection authorities during the research phase, rather than after prototype development, is likely to substantially reduce the gap between proof-of-concept and operational deployment.

## 5 Limitations

This review has several limitations worth stating directly. The search covered PubMed, Scopus, and Web of Science only, IEEE Xplore and ACM Digital Library were not searched at either stage. Their exclusion likely underrepresents engineering and HCI contributions. Future reviews in this domain should include these databases.

Only English-language articles were included, which may underrepresent research from non-Anglophone institutions, a relevant gap given the volume of ATC vigilance research from China identified in this review.

The predominance of simulator-based studies and the heterogeneity of ground truth definitions made cross-study comparison difficult and ruled out meta-analysis. One source initially retrieved (Li et al., 2026) is an SSRN preprint that has not completed peer review; consistent with Frontiers in Neuroergonomics policy, it was excluded from the final synthesis. The review was not pre-registered on PROSPERO prior to data collection, which leaves it without protection against outcome reporting bias.

## 6 Conclusions

Thirty-four studies show that vigilance in ATC is measurable through physiological and behavioral signals, and that machine learning models can predict decrements with promising accuracy under controlled conditions, though the absence of meta-analysis and the predominance of small, within-subject, simulator-based designs limit how far these accuracy figures can be generalized. Three observations are noted here as descriptive patterns rather than confirmed effects: RDT environments introduce underload and out-of-the-loop risks that existing monitoring approaches do not adequately address; UAM contexts represent an emerging operational domain not yet represented in the vigilance monitoring literature; and within individual studies, designs incorporating multimodal fusion or advanced architectures such as spatio-temporal graph networks and CNN-LSTMs were sometimes associated with higher reported accuracy values than single-modality or simpler comparators used within the same study. These within-study observations do not constitute evidence of generalizable superiority across the literature: the comparison rests entirely on narrative synthesis across highly heterogeneous designs, small and predominantly within-subject samples, simulator-based settings, and non-comparable outcome metrics, and no meta-analytic pooling was possible. Higher reported accuracy in any single study is not evidence of better generalisability, broader applicability, or superior capture of vigilance dynamics.

The distance between these results and operational deployment is still large. The distinction between proof-of-concept detection and an operationally deployable monitoring system has not been consistently maintained in the reviewed literature. Bridging that gap requires individual calibration, closed-loop intervention design, outputs that operational personnel can actually interpret, field validation at scale, and regulatory engagement, pursued in parallel, not as a sequential checklist, by anyone building a system for live ATC operations.

## Data Availability

The original contributions presented in the study are included in the article/Supplementary material, further inquiries can be directed to the corresponding author.

## Supplementary material

Supplementary Table S1 Completed PRISMA 2020 checklist, cross-referencing each of the 27 reporting items to the corresponding section, table, or figure in the manuscript where it is addressed.
