# Supplementary material for: Vigilance decrements in air traffic control as an aviation safety risk: a systematic review of modeling and prediction approaches
Source: Front Neuroergon. 2026 Aug 13;7:1874304. doi: 10.3389/fnrgo.2026.1874304 (PMC13518319; doi:10.3389/fnrgo.2026.1874304)
Supplement: Supplementary file 1 [file Table_1.docx]

# PRISMA 2020 Checklist

| TITLE | | | |
| --- | --- | --- | --- |
| Title | 1 | Identify the report as a systematic review. | **Reported.** Title page, “Vigilance Decrements in Air Traffic Control as an Aviation Safety Risk: A Systematic Review of Modeling and Prediction Approaches.” |
| ABSTRACT | | | |
| Abstract | 2 | See the PRISMA 2020 for Abstracts checklist. | **Reported.** Abstract states objective, databases searched (three), search dates (initial Jan 2025; forward update Feb 2026), pre-specified inclusion criteria, number of included studies (34), the modalities and model classes synthesized, and the real-time-deployment appraisal lens. |
| INTRODUCTION | | | |
| Rationale | 3 | Describe the rationale for the review in the context of existing knowledge. | **Reported.** Section 1 (Introduction), establishes vigilance failure as an ATC safety problem, the bidirectional load effect, the lab-to-floor gap, and why vigilance is the organising construct (three-reason paragraph). |
| Objectives | 4 | Provide an explicit statement of the objective(s) or question(s) the review addresses. | **Reported.** Section 1, final paragraph (four objectives) and Section 2.1 (review question + PICOS framework). |
| METHODS | | | |
| Eligibility criteria | 5 | Specify the inclusion and exclusion criteria for the review and how studies were grouped for the syntheses. | **Reported.** Section 2.3 and Table 1 (inclusion/exclusion criteria). Grouping for synthesis: two-tier evidence framework (Section 4.1) and characteristic tabulation in Tables 3 and 4. |
| Information sources | 6 | Specify all databases, registers, websites, organisations, reference lists and other sources searched or consulted to identify studies. Specify the date when each source was last searched or consulted. | **Reported.** Section 2.2, PubMed, Scopus, Web of Science Core Collection; initial search dates (PubMed 15 Jan 2025, Scopus 16 Jan 2025, WoS 17 Jan 2025) and forward citation update dates (25–27 Feb 2026), plus citation tracking from included studies. |
| Search strategy | 7 | Present the full search strategies for all databases, registers and websites, including any filters and limits used. | **Reported.** Section 2.2.1, full verbatim Boolean strings for all three databases with field codes, language and document-type filters, and date limits; records retrieved per database stated. |
| Selection process | 8 | Specify the methods used to decide whether a study met the inclusion criteria of the review, including how many reviewers screened each record and each report retrieved, whether they worked independently, and if applicable, details of automation tools used in the process. | **Reported.** Section 2.3, title/abstract screening by MA with a 20% independent sample by AM (κ = 0.81); full-text review independently by MA and JEA; disagreements by consensus/arbitration (JEA, then AM). No automation tools used. |
| Data collection process | 9 | Specify the methods used to collect data from reports, including how many reviewers collected data from each report, whether they worked independently, any processes for obtaining or confirming data from study investigators, and if applicable, details of automation tools used in the process. | **Reported.** Section 2.5, a structured extraction sheet applied uniformly across all 34 studies. Data were taken from published reports; no contact with study investigators and no automation tools. |
| Data items | 10a | List and define all outcomes for which data were sought. Specify whether all results that were compatible with each outcome domain in each study were sought, and if not, the methods used to decide which results to collect. | **Reported.** Section 2.1 (Outcomes: primary, vigilance classification accuracy, detection sensitivity, generalizability; secondary, sensor practicality, calibration requirements, closed-loop design) and Section 2.5 (metrics: accuracy, sensitivity, specificity, F1, false-alarm rate where reported). Best reported performance per study was extracted. |
| Data items | 10b | List and define all other variables for which data were sought (e.g. participant and intervention characteristics, funding sources). Describe any assumptions made about any missing or unclear information. | **Reported.** Section 2.5, study parameters, participant N and characteristics, task setting and duration, paradigm, vigilance manipulation, modality, features, modeling approach, validation strategy, and setting. Where validation type was not stated, it was inferred from the experimental description (noted in Table 4) |
| Study risk of bias assessment | 11 | Specify the methods used to assess risk of bias in the included studies, including details of the tool(s) used, how many reviewers assessed each study and whether they worked independently, and if applicable, details of automation tools used in the process. | **Reported.** Sections 2.4 and 2.4.1, domain-adapted six-domain appraisal checklist with sub-items and Y/P/N/NA thresholds; assessed independently by MA and JEA (κ = 0.76), disagreements resolved with AM. No automation tools. |
| Effect measures | 12 | Specify for each outcome the effect measure(s) (e.g. risk ratio, mean difference) used in the synthesis or presentation of results. | **Reported.** Sections 2.5 and 3.4, performance measures used: classification accuracy, sensitivity, specificity, F1-score, false-alarm rate, mAP, and R². No pooled effect measure; synthesis is narrative. |
| Synthesis methods | 13a | Describe the processes used to decide which studies were eligible for each synthesis (e.g. tabulating study characteristics and comparing against the planned groups). | **Reported.** Sections 3.4 and 4.1, studies tabulated by characteristics (Tables 3, 4) and assigned to Tier 1 (proof-of-concept) or Tier 2 (stronger external validity) for synthesis. |
| Synthesis methods | 13b | Describe any methods required to prepare the data for presentation or synthesis, such as handling of missing summary statistics, or data conversions. | **Reported.** Sections 2.5 and 3.4, metrics tabulated as reported in source papers; missing operational metrics flagged as “N/R” and accuracy retained. No statistical conversion or imputation was performed. |
| Synthesis methods | 13c | Describe any methods used to tabulate or visually display results of individual studies and syntheses. | **Reported.** Section 3, Table 3 (distribution of study characteristics) and Table 4 (per-study ML/DL results with N, validation, setting, metrics, evidence tier); narrative synthesis text in Sections 3.1–3.4. |
| Synthesis methods | 13d | Describe any methods used to synthesize results and provide a rationale for the choice(s). If meta-analysis was performed, describe the model(s), heterogeneity methods, and software. | **Reported.** Section 3.4, narrative/structured synthesis used; meta-analysis explicitly not conducted owing to heterogeneity in design, samples, task fidelity, modalities, and validation strategy. Rationale stated. |
| Synthesis methods | 13e | Describe any methods used to explore possible causes of heterogeneity among study results (e.g. subgroup analysis, meta-regression). | **Reported.** Sections 3.4, 4.1–4.3, heterogeneity examined qualitatively via the two-tier framework and structured comparison across sample size, validation design, setting (simulator vs. field), and ground-truth definition. No meta-regression (no meta-analysis). |
| Synthesis methods | 13f | Describe any sensitivity analyses conducted to assess robustness of the synthesized results. | **Not applicable.** No meta-analysis was performed, so quantitative sensitivity analyses were not conducted (see Section 3.4). |
| Reporting bias assessment | 14 | Describe any methods used to assess risk of bias due to missing results in a synthesis (arising from reporting biases). | **Not applicable / acknowledged.** No quantitative synthesis was conducted, so funnel-plot / small-study assessment was not applicable. The absence of prospective PROSPERO registration, which reduces protection against outcome reporting bias, is disclosed in Sections 2.6 and 5. |
| Certainty assessment | 15 | Describe any methods used to assess certainty (or confidence) in the body of evidence for an outcome. | **Reported (adapted).** Certainty addressed through the six-domain quality appraisal (Sections 2.4–2.4.2, Table 2) and the Tier 1 / Tier 2 evidence framework (Section 4.1). A formal GRADE assessment was not applied, given the non-comparative study designs. |
| RESULTS | | | |
| Study selection | 16a | Describe the results of the search and selection process, from the number of records identified to the number of studies included, ideally using a flow diagram. | **Reported.** Section 2.3 and Figure 1 (PRISMA 2020 flow diagram): 850 records identified, 550 after deduplication, plus 47 from the forward update (12 retained, 8 to full text); 501 excluded at title/abstract; 57 full texts assessed, 23 excluded; 34 included. |
| Study selection | 16b | Cite studies that might appear to meet the inclusion criteria, but which were excluded, and explain why they were excluded. | **Reported.** Section 5, SSRN preprint [51] excluded for not completing peer review (per journal policy). Figure 1 gives full-text exclusion counts by reason (insufficient ATC/aviation focus n=6; no vigilance measure n=4; insufficient detail/inaccessible n=5; preprint n=1; other criteria n=7). |
| Study characteristics | 17 | Cite each included study and present its characteristics. | **Reported.** Section 3.1 and Tables 3 and 4, each included study cited with population, setting, modality, model, sample size, validation, and performance. |
| Risk of bias in studies | 18 | Present assessments of risk of bias for each included study. | **Reported.** Table 2 (Section 2.4.2), per-study domain-level ratings (D1–D6) and High/Medium/Low classification for all 34 studies. |
| Results of individual studies | 19 | For all outcomes, present for each study summary statistics and an effect estimate with its precision, ideally using structured tables or plots. | **Reported (as available).** Table 4, best reported performance per study with N, validation type, setting, and operational metrics where the source paper reported them. Precision intervals were generally not reported in primary studies; this is flagged as “N/R”. |
| Results of syntheses | 20a | For each synthesis, briefly summarise the characteristics and risk of bias among contributing studies. | **Reported.** Sections 3.4 and 4.1 with Tables 2 and 4, contributing studies summarised by tier, with quality classification carried into interpretation. |
| Results of syntheses | 20b | Present results of all statistical syntheses conducted. If meta-analysis was done, present the summary estimate, its precision, and heterogeneity measures. | **Reported (narrative).** Section 3.4, reported accuracy ranged 79–99%; multimodal-fusion and CNN-LSTM / spatio-temporal-graph models trended higher, with the caveat of small samples and within-subject validation. No pooled estimate (no meta-analysis). |
| Results of syntheses | 20c | Present results of all investigations of possible causes of heterogeneity among study results. | **Reported.** Sections 4.1–4.3, heterogeneity attributed to sample size and validation design (4.2), simulator vs. operational fidelity (4.3), and divergent ground-truth definitions (4.1). |
| Results of syntheses | 20d | Present results of all sensitivity analyses conducted to assess the robustness of the synthesized results. | **Not applicable.** No meta-analysis was performed; quantitative sensitivity analyses were therefore not conducted. |
| Reporting biases | 21 | Present assessments of risk of bias due to missing results (arising from reporting biases) for each synthesis assessed. | **Not applicable / acknowledged.** No quantitative synthesis; formal reporting-bias assessment not applicable. Limitation acknowledged in Sections 2.6 and 5 (non-registration). |
| Certainty of evidence | 22 | Present assessments of certainty (or confidence) in the body of evidence for each outcome assessed. | **Reported (adapted).** Section 4.1 with Tables 2 and 4, confidence expressed through quality classification and the Tier 1 / Tier 2 framework. On source review, no included study provided combined field and cross-subject evidence; deployability is therefore discussed as an aspiration, with the cross-subject studies [29, 50] offering the strongest available external validity. |
| DISCUSSION | | | |
| Discussion | 23a | Provide a general interpretation of the results in the context of other evidence. | **Reported.** Section 4.1, cross-study synthesis and primary implications for aviation safety; four operational-deployability criteria set against the evidence. |
| Discussion | 23b | Discuss any limitations of the evidence included in the review. | **Reported.** Sections 4.2 (small samples / power / overfitting), 4.3 (simulator vs. real-world fidelity), and 4.4 (measurement-technology / dry-electrode constraints). |
| Discussion | 23c | Discuss any limitations of the review processes used. | **Reported.** Section 5, omission of IEEE Xplore and ACM Digital Library, English-only restriction, heterogeneity precluding meta-analysis, and absence of PROSPERO registration. |
| Discussion | 23d | Discuss implications of the results for practice, policy, and future research. | **Reported.** Section 4.5 (closed-loop design, explainability, individual calibration, field validation, regulatory/ethical integration) and Section 6 (Conclusions). |
| OTHER INFORMATION | | | |
| Registration and protocol | 24a | Provide registration information for the review, including register name and registration number, or state that the review was not registered. | **Reported.** Sections 2.6 and 5, the review was not registered on PROSPERO; this is stated explicitly as a limitation. |
| Registration and protocol | 24b | Indicate where the review protocol can be accessed, or state that a protocol was not prepared. | **Reported.** Section 2.6, no separate registered protocol was prepared in advance; the methods (search strategy, eligibility, appraisal) are documented in Sections 2.2–2.4. |
| Registration and protocol | 24c | Describe and explain any amendments to information provided at registration or in the protocol. | **Reported.** Section 2.2, the forward citation update (through Feb 2026) is disclosed as an amendment to the original Jan 2025 search, with the same eligibility criteria applied. |
| Support | 25 | Describe sources of financial or non-financial support for the review, and the role of the funders or sponsors. | **Reported.** Funding statement, “This research received no external funding.” |
| Competing interests | 26 | Declare any competing interests of review authors. | **Reported.** Conflicts of Interest statement, “The authors declare no conflicts of interest.” |
| Availability of data, code and other materials | 27 | Report which of the following are publicly available and where: data collection forms; data extracted from included studies; data used for analyses; analytic code; other materials. | **Reported.** Data Availability Statement, no new data were generated; all sources are published peer-reviewed articles. Full search strings (Section 2.2.1), appraisal checklist (Section 2.4.1), per-study ratings (Table 2), and extracted results (Table 4) are provided in-manuscript. No separate analytic code was used. |

Abbreviations: ATC, air traffic control; κ, Cohen’s kappa; ML/DL, machine learning / deep learning; N/R, not reported; PROSPERO, International Prospective Register of Systematic Reviews.
